# Editorialets

**Published:** 1908-03

**Authors:** 


### Editorialets.

DR. GEO. R. TABOR, of Dallas, late State Health Officer of Texas, has been elected by the Executive Committee President of the American International Congress on Tuberculosis. The Congress will hold its fifth biennial session in Chicago in June next.

DR. DERO EUGENE SEAY, of Dallas, was married on Tuesday, March 3d, at 6 o'clock, at the First Presbyterian Church, Dallas, Texas, to Miss Pauline Adrienne Bolanz, of that city. Thanks for cards.

BEGINNING with the April issue, the *Electro-Therapeutist*, formerly published by H. C. Bennett, M. D., of Lima, Ohio, will be consolidated with *Albright's Office Practitioner*, Philadelphia. There will be no change in the title, policy or management of the latter journal, the only change being an increase of the number of pages.

INTERNATIONAL MEDICAL ASSOCIATION (MEXICO).—At the recent meeting of this Association held in Monterey, Mexico, the following officers were elected:

President, Dr. W. R. Jamieson, Torreon, Coahuila; First Vice-President, Dr. R. H. L. Bibb, Saltillo, Coahuila; Second Vice-President, Dr. Mary L. Burnham, Monterey, N. L.; Secretary and Treasurer, Dr. J. S. Steele, Monterey, N. L.; Censor for three years' term, Dr. R. H. L. Bibb, Saltillo, Coahuila; Editors of the Annual, Drs. Jamieson and Robinson, Torreon, Coahuila.

Next meeting at Tampico, January, 1909.

DR. T. J. TYNER died in Washington City, February 8th, ult., after a protracted illness. Dr. Tyner was long a resident of San Antonio, and later of Austin. He removed to Washington in 1894, and engaged in the practice of his specialty—diseases of the eye. He was a learned and popular physician, greatly loved and admired by a wide circle of friends, not only in Texas, but throughout the United States, who will regret to hear of his early death. He was a native of Tennessee and a graduate of the College of Physicians and Surgeons, Keokuk, Iowa, 1875. He leaves a wife, but no childern. Mrs. Tyner was a Memphis lady, well known in that city.

MEATOX is not an extract of beef. It is not a predigested food. It is the fiber of lean beef, sterilized and dried. It is free from preservatives but keeps fresh indefinitely in unsealed containers. It is more easily digested than egg albumen. It is palatable. It can be administered either in its original granulated form or else mixed with soft boiled eggs, hominy, oat meal, soup, broth, or any kind of food which may agree best with the patient. One ounce of Meatox is equal to about five ounces of the best lean beef for its nutritive value.

---

TRAINING IN MEDICAL ORGANIZATION.—The students of the University of Pennsylvania Medical School have formed an organization, the purpose of which is to acquaint the undergraduates with the workings of the American Medical Association, after which it is very closely modeled. The various student societies take the place of the State organization and elect members to a house of delegates, which transacts all the business of the Association. An annual meeting is held at which papers are read by chosen members, thus encouraging original research and a scientific spirit. house of delegates, which transacts all the business of the association of the University of Pennsylvania, and already has over two hundred and fifty members.

